# Revealing parasite influence in metabolic pathways in Apicomplexa infected patients

**DOI:** 10.1186/1471-2105-11-S11-S13

**Published:** 2010-12-14

**Authors:** Tao Xu, Jie Ping, Yao Yu, Fudong Yu, Yongtao Yu, Pei Hao, Xuan Li

**Affiliations:** 1 College of life science and biotechnology, Shanghai Jiao Tong University, Shanghai, 200240, China; 2 Key Lab of Systems Biology/Key Laboratory of Synthetic Biology, Shanghai Institutes for Biological Sciences, Chinese Academy of Sciences, Shanghai, 200031, China; 3 Shanghai Center for Bioinformation Technology, 100 Qinzhou Road, Shanghai, 200235, China

## Abstract

**Background:**

As an obligate intracellular parasite, Apicomplexa interacts with the host in the special living environment, competing for energy and nutrients from the host cells by manipulating the host metabolism. Previous studies of host-parasite interaction mainly focused on using cellular and biochemical methods to investigate molecular functions in metabolic pathways of parasite infected hosts. Computational approaches taking advantage of high-throughput biological data and topology of metabolic pathways have a great potential in revealing the details and mechanism of parasites-to-host interactions. A new analytical method was designed in this work to study host-parasite interactions in human cells infected with Plasmodium falciparum and Cryptosporidium parvum.

**Results:**

We introduced a new method that analyzes the host metabolic pathways in divided parts: host specific subpathways and host-parasite common subpathways. Upon analysis on gene expression data from cells infected by Plasmodium falciparum or Cryptosporidium parvum, we found: (i) six host-parasite common subpathways and four host specific subpathways were significantly altered in plasmodium infected human cells; (ii) plasmodium utilized fatty acid biosynthesis and elongation, and Pantothenate and CoA biosynthesis to obtain nutrients from host environment; (iii) in Cryptosporidium parvum infected cells, most of the host-parasite common enzymes were down-regulated, whereas the host specific enzymes up-regulated; (iv) the down-regulation of common subpathways in host cells might be caused by competition for the substrates and up-regulation of host specific subpathways may be stimulated by parasite infection.

**Conclusion:**

Results demonstrated a significantly coordinated expression pattern between the two groups of subpathways. The method helped expose the impact of parasite infection on host cell metabolism, which was previously concealed in the pathway enrichment analysis. Our approach revealed detailed subpathways and metabolic information are important to the symbiosis in two kinds of the apicomplex parasites, and highlighted its significance in research and understanding of parasite-host interactions.

## Background

Living organisms gain energy from and discharge wastes to their environment. In the case of obligate intracellular parasites, living environment changes with the internal biochemical state of hosts. The need of intracellular parasites to retrieve nutrients and fulfill their energy requirements is achieved by manipulating the host metabolism. Such parasitic relationships, as well as other forms of species-to-species interactions, set the selective pressures to shape the evolution of each species [1]. Therefore the metabolic networks are markedly impacted, e.g. many intracellular parasites have lost a substantial number of genes related to biosynthetic functions, and depend on the hosts for nutrients that they can not produce themselves[2,3].

Many different approaches have been developed to study the possible interactions of host and parasite in metabolic pathways. Some traditional cellular and biochemical methods investigating protein or molecular functions in metabolic pathways were used in previous studies [4,5]. Computational approaches using high throughput biological data would have great potential in revealing parasites-to-host interactions. Topology of metabolic pathways can be used to evaluate the biosynthetic support for a given pair of species by identifying the set of exogenously acquired compounds in the parasite metabolic network. Metabolites that parasites gained from the host can be predicted on the basis of topological structure of the metabolic pathway. It was shown previously that parasite tends to reduce their own metabolic pathways to make the metabolism most efficient, and utilize the metabolites from the host cells. The adaptation to parasitic environment, i.e. obtaining metabolites from their hosts, represents the life habit of these microorganisms [6,7]. In such cases, it was hypothesized that there would be an inhibitive effect on the host genes which are downstream of metabolites that parasites need, but an increase on the genes that produce such metabolites. Thus, the infection of parasite would cause a biochemical imbalance in host cells [8]. Our study is focused on a type of special protozoan, apicomplexa, which interacts with its host via its unique apical organelles, apicolast. This kind of parasite has a complex life cycle. After attachment and orientation on the erythrocyte surface, apicomplexa produce the small endocytic vacuole which accommodates the parasite in the feeding stage[9]. This organelle is vital for the nutrient obtaining, reproducing, and other aspects of the parasite. It facilitates the direct protein-protein interactions and nutrient transportation between host and parasite [10,11]. In the current study, the expressions of genes that encoded the related enzymes are examined. We hoped to evaluate the impact of infection on the expression of related genes based on the topological information of metabolic pathways, and the host-parasite ortholog gene pairs. The biological pathways that were involved in the nourishing relationship between host and parasite were analyzed to reveal the parasite-host interactions in greater details.

## Methods

### Metabolic pathway information and expression data

A study on malaria previously reported by Ockenhouse, C. F. et al. provided the expression data of epithelial blood cell from acute infected patients and uninfected persons. The data was obtained from the GEO (GDS2362). Time series data were as retrieved from a work on the expression of Cryptosporidium parvum infected HCT-8 ileocecal epithelial cells at various time points, including 6 time points, up to 72 hours after infection with Cryptosporidium parvum (GDS2240). KEGG orthology (KO) information and pathway information of both host’s and parasite’s genes were downloaded from the Kyoto Encyclopedia of Genes and Genomes (KEGG) [12]. The genes with ortholog in both parasite and the host were defined as host-parasite ortholog genes. Genes owned by host alone (without ortholog in parasite) were defined as host-specific genes.

### Identification of differentially expressed genes (DEGs)

The dataset of GDS2362 was obtained from Affymetrix Human Genome U95 Version 2 Array (GPL8300), and that of GDS2362 from Affymetrix Human Genome U133A Array (GPL96). We used the BioConductor package ‘affy’ to perform the data preprocessing and normalization[13]. Both datasets were background corrected and normalized using Robust Multichip Average (RMA) algorithm in ‘affy’ package. Genes with absolute log_2_-fold changes greater than 1.5 and a *P* value less than 0.05 were considered significantly differentially expressed genes. A list of differentially expressed genes, their fold changes, and P values of t-test at different time points is provided in the additional files 1,2,3.

### Definition of host-parasite ortholog genes

Original KEGG-defined ortholog genes were used in our analysis without any changes. KEGG provides a list of ortholog genes, which were downloaded from KEGG web site (http://www.genome.jp/kegg/). We identified the host-parasite ortholog gene pairs as: genes from the genomes of both host and parasite that belong to a group of ortholog genes in KEGG (one KO group).

### Enrichment analysis of DEGs on metabolic pathways

The differentially expressed genes were classified into two groups, according to their intersection with the host-parasite ortholog genes and host-specific genes separately, which were named ortholog-DEG and nonortholog-DEG. The enrichment analysis was used to detect the significance of alternation on each metabolic pathway. [14] For each differentially expressed gene groups, we use the hyper geometric distribution to calculate the probability that k out of n genes in one pathway differentially expressed, while m genes in the microarray experiment differentially expressed. P-value could be calculated from the hyper geometric test given in (1), while N indicates number of genes on the chip.(1)

Bonferroni correction was performed to adjust the P-values, l represents the number of comparisons in the test.(2)

### The pathway regulation pattern in different gene sets

The expression states of a particular KEGG pathway were investigated according to the up- or down-regulation of the two DEG sets, as described in Table 1. Similarly, we used the enrichment analysis described above to test if either set of genes were significantly up or down-regulated, considering the corresponding regulation of all genes on the chip as the background, where N stands for the total number of genes on the chip and m indicates the genes that were up- or down- regulated among all genes. Given the total number of genes in a pathway and genes which were up-regulated, the probability of observing k or more sequences for genes in this pathway can be calculated by the formula (1). The Bonferroni correction was used to adjust the p-values for gene enrichment analysis based on the hyper geometric distribution.

**Table 1:** The host-parasite common subpathways significantly up- or down- regulated in plasmodium infected human blood cells.

KEGG_ID	description	Hits/Total
**common.up**		
hsa00680*	Methane metabolism	2/3

**common.down**		
hsa00072**	Synthesis and degradation of ketone bodies	2/2
hsa00591**	Linoleic acid metabolism	1/1
hsa00770**	Pantothenate and CoA biosynthesis	1/1
hsa00900**	Terpenoid backbone biosynthesis	4/4

**specific.up**		
hsa00061*	Fatty acid biosynthesis	1/2
hsa00062*	Fatty acid elongation in mitochondria	3/8
hsa00330*	Arginine and proline metabolism	10/38
hsa00410*	beta-Alanine metabolism	6/19
hsa00564*	Glycerophospholipid metabolism	8/32
hsa00592*	alpha-Linolenic acid metabolism	4/13
hsa00640*	Propanoate metabolism	5/16
hsa00670*	One carbon pool by folate	3/9
hsa00780*	Biotin metabolism	2/4
hsa00903*	Limonene and pinene degradation	4/14
hsa01040*	Biosynthesis of unsaturated fatty acids	4/14
hsa00290**	Valine, leucine and isoleucine biosynthesis	2/2

**specific.down**		
hsa00300**	Lysine biosynthesis	2/2
hsa00740**	Riboflavin metabolism	6/6
hsa00790**	Folate biosynthesis	6/7

Hits/Total indicates the number of genes significantly altered among all the genes in the subpathway. (* p-value≤0.1; ** p-value≤0.05)

## Results

### Analysis of plasmodium infected human cell

Firstly, we tried to find which genes are differentially expressed after infection. Using false discovery rate (FDR) less than 0.01, we found a group of genes whose expressions were significantly altered when comparing the epithelial blood expression profile from acutely infected patient with from uninfected people. After that, we performed enrichment analysis to detect significantly altered expression pathways using the hyper geometric method (see *Method),* which may reveal significance of gene-set wise alternation[15]. The result indicates two pathways (KEGG ID hsa00062: Fatty acid elongation in mitochondria, hsa00410: beta-Alanine metabolism) were significantly altered (FDR<=0.01) in the samples obtained from patients having clinically apparent malaria.

Then we separated pathways into host specific pathways and host-parasite common pathways. According to the KEGG orthology (KO) information, we first identified 1357 genes for which host-parasite ortholog pairs exist, and 8092 genes which were found only in host. We defined a subpathway consisting of ortholog pairs as host-parasite common subpathway, and a subpathway consisting of host only genes as a host specific subpathway. We subsequently performed enrichment test on each subpathway. Filtering out the subpathway s that contain only one gene, the result showed 6 host-parasite common subpathways and 4 host specific subpathways were significantly altered (Additional File 1).

**Figure 1:**
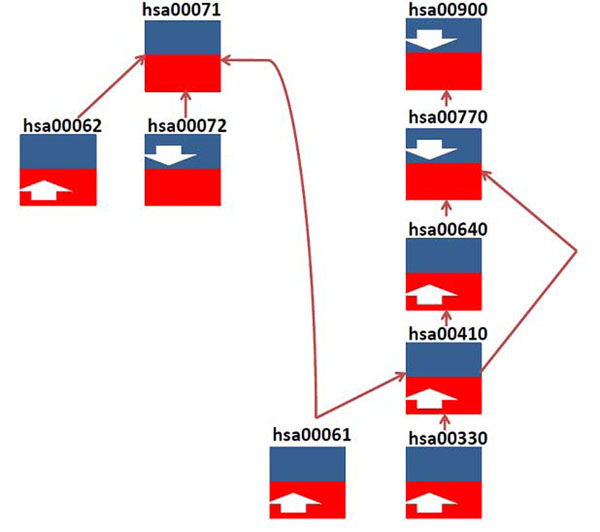
**Pathway regulation in Plasmodium infected human periphery blood**. Each pathway was divided into two subpathways according to ortholog genes defined in Kyoto Encyclopedia of Genes and Genomes. Each box represents a pathway. The blue part denotes host-parasite ortholog genes constituted subpathway, which is named host-parasite common subpathway; the red part is constituted by host genes which have no ortholog in parasite, and is named host-specific subppathway. In each subpathway, the arrow indicates if the subpathway is significantly up- or down- regulated (p-value≤0.05 after bonferroni correction as significant; p-value≤0.1 after bonferroni correction provided to give more complete information on linked pathways/sub-pathways). We showed here the regulation pattern of pathways related to metabolism of fatty acid (KEGG ID hsa00061, hsa00062 and has00071) and pantothenate (KEGG ID hsa00770).

Previously differentially expressed genes were classified to up-regulated or down-regulated gene sets in order to analyze the response of host cells after infection of pathogen. In this study, we further divided them into four different sub-sets according to their up- or down-regulation. We did enrichment test on each of these four sets, and found several pathways that had either host-parasite common or host specific subpathways showing an up- or down-regulation pattern (Table 1). All the test p-values were adjusted using Bonferroni correction. To analyze data more comprehensively we provided the results with multiple thresholds: p-value≤0.05, and p-value≤0.1. Furthermore, we found there exist correlated changes among these subpathways, and more importantly we identified subpathways that plasmodium utilized to obtain nutrients for its own development (Figure 1). They include Fatty acid biosynthesis and elongation (KEGG ID hsa00061 and hsa00062), and Pantothenate and CoA biosynthesis (KEGG ID hsa00770).

**Figure 2:**
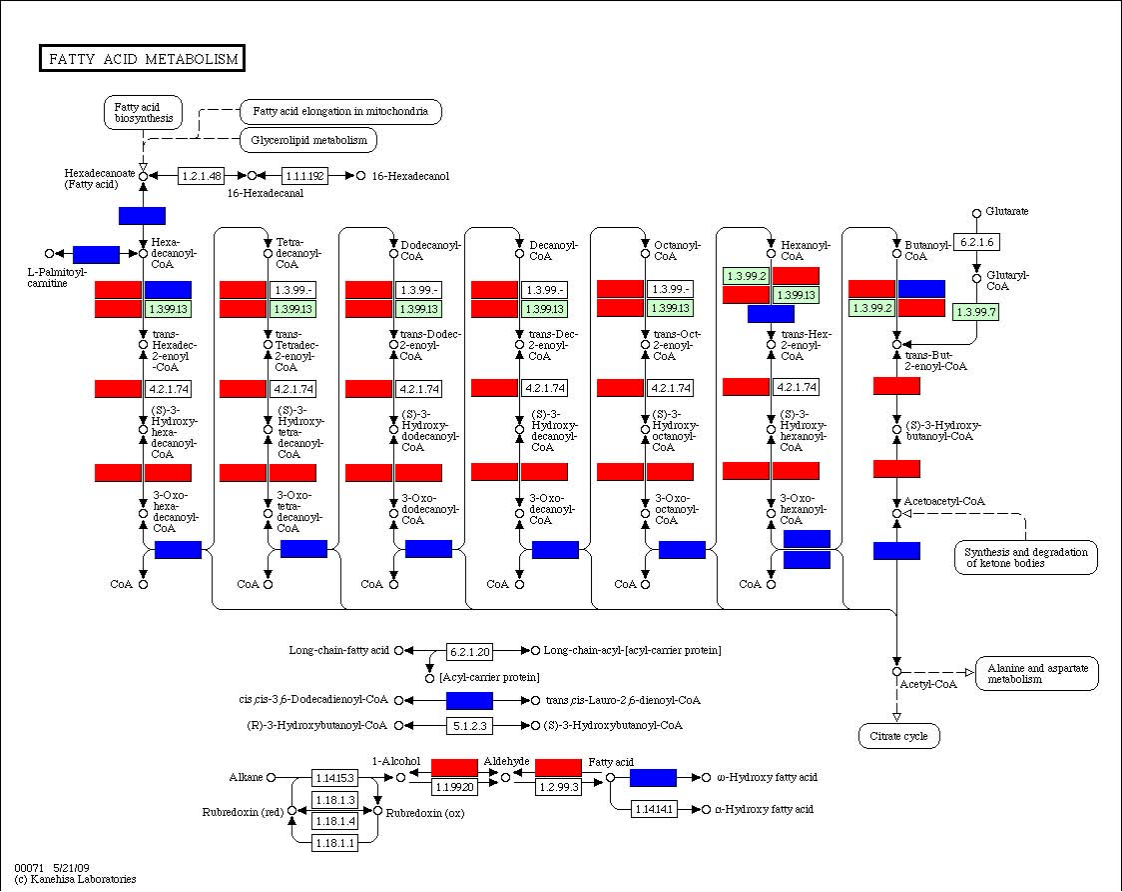
**The regulation of genes in the pathway of fatty acid metabolism (KEGG ID hsa00071)**. A red rectangle indicates gene expression that was up-regulated significantly after infection of plasmodium and blue indicates down-regulation.

As shown in Figure 1, host specific subpathway of both fatty acid biosynthesis and elongation were up-regulated. Yet, another pathway (KEGG ID hsa00072) which is also the upstream of fatty acid metabolism in the human cell has a significant down-regulation pattern in its host-parasite common subpathway. The fatty acid elongation (KEGG ID hsa00062) has 3 out of 8 genes in the host-specific sub-path which were significantly up-regulated. The situation in fatty acid metabolism (KEGG ID hsa00071) would be a little complicated; 7 out of 32 genes were up-regulated while 10 genes down-regulated in the host-specific part of fatty acid metabolism. And in the host-parasite common part, there were 3 out of 7 genes up- regulated. Neither part of its pathway show a significant up- nor down- regulation pattern. Structural information of the pathway might be considered to evaluate the process. We used a graphic method to map regulation information on the KEGG pathway and further the analysis in a higher resolution (Figure 2). We found genes which are down-stream of Synthesis and Degradation of Ketone Body (KEGG ID hsa00072), fatty acid elongation and biosynthesis (KEGG ID hsa00061 and hsa00062) were down-regulated, while most of the genes in the middle of the pathway were up-regulated significantly. Comparing the fatty acid metabolic pathway (KEGG ID cpv00071) of Plasmodium, most of the host-parasite common enzymes were down-regulated in the pathway, while the host specific enzyme up-regulated. In fact, recent study has shown that Plasmodium parasites rely on de novo fatty acid synthesis only for liver-stage development. But as the infection on patients became clinically apparent, which is a symptomatic stage of malaria, Plasmodium scavenges fatty acids mainly from its host blood cell [16].

Pantothenate is another important nutrient for plasmodium. Here, we also detected significant alternation in the pathway of Pantothenate and CoA biosynthesis (KEGG ID hsa00770). P. falciparum requires an extracellular supply of pantothenate to support its proliferation during the erythrocytic stage of its development in humans [17]. We found host-specific part of its upstream pathways, such as Propanoate metabolism (KEGG ID hsa00640), beta-Alanine metabolism (KEGG ID hsa00410), and Arginine and proline metabolism (KEGG ID hsa00330) all significantly up-regulated. But the host-parasite common part of Pantothenate and CoA biosynthesis (KEGG ID hsa00770) and its downstream pathway Terpenoid backbone biosynthesis (KEGG ID hsa00900) both significantly down-regulated. Based on this result, we hypothesized that some compound in these pathway being scavenged by plasmodium from host cell. One recent finding by Kellen L. Olszewski et.al. provided support to our hypothesis. Their study showed that the extracellular arginine in infected host cells differed from uninfected cells irrespective of the developmental stage. The Plasmodium converts arginine to ornithine and causes depletion in the host cell which is related to the human malarial hypoargininemia associated with cerebral malaria pathogenesis[18].

Other detected pathways that have their subpathways up or down-regulated also show important aspects of plasmodium during its development. The host-parasite common subpathway of Linoleic acid metabolism (KEGG ID hsa00591) was gene enriched and showed a down-regulation pattern. Linoleic acid is well known for its promotion of beta-hematin formation in vitro. Previous study suggests that linoleic acid or diglyceride containing dilinoleolyglycerol plays the critical role in promoting FP polymerization in malaria parasites.[19]

### Analysis of Cryptosporidium parvum and its infection in different time points

**Figure 3:**
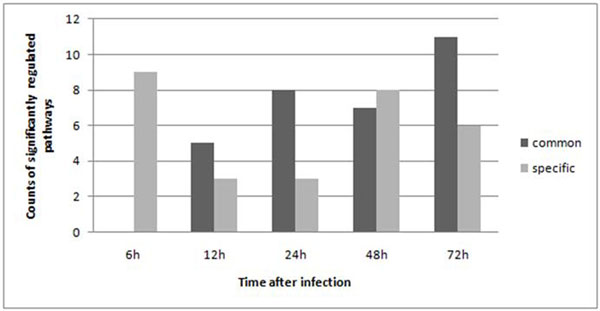
**Histogram of number of subpathways which have significantly alternation in either host-parasite common subpathway or host-specific subpathway.** “Common” denotes host-parasite common subpathways that were significantly altered. “Specific” denotes host-specific subpathways that were significantly altered.

We use the same method as above analysis to detect DEGs (detail see *Method*), based on data from a previous study on human cell expression response[20]. Result showed, during the infection, the amount of differentially expressed genes varies (9%~14%). At 6 hours post infection it had its minimum as 780, which is 8.8% of all genes on the chip. But, as the infection time extended, the differential expressed gene number grew. At 12, 24, 48, and 72 hours after infection there were 1092, 1160, 1106 and 1070 DEGs, respectively. Genes were divided into two sets as we did in the above study on Plasmodium, but in different time points. For each time point, there are always more pathways significantly altered in their host parasite common part (Figure 3), but the case of 6 hours after infection is an exception. Investigating the time line, we found that generally speaking, as the infection time expended, more metabolic pathways show a significant DEG enrichment. That indicted the effect of parasite infection on ortholog genes was magnified during infection. We gave a list of the pathways that were significantly alternated (p-value≤0.05) in either of its parts during the infection (see Additional file 3).

**Figure 4:**
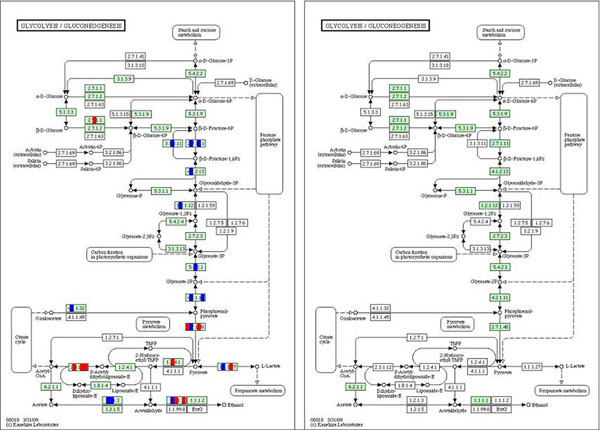
**The Glycolysis and Gluconeogenesis pathway of human (left) and C.parvum (right)**. The host gene expression response was indicated for each gene in the pathway. Each rectangle, standing for a enzyme in the pathway, is divided into five parts that represent five infection time points: 6, 12, 24, 48, 72hr after infection. Red color indicates an up-regulation of this enzyme at the corresponding time point, and blue indicates a down-regulation. The green background boxes indicate the ortholog genes shared by human host cell and C.parvum.

We found that pathways having a different regulation pattern between host-parasite ortholog genes and host specific genes, were parasite nutrient dependent. First is the pathway of Glycolysis/Gluconeogenesis for example, shown in Figure 4. The host-parasite homologous genes in the host metabolic pathway have a pattern of universally, and significantly as well, down-regulation. However, for the host genes that do not have a homolog in parasite genome, a significant number of the genes are up-regulated. According to the previous study made by Abrahamsen [21] on the genome of the parasite, Cryptosporidium was suspected to have an incomplete gluconeogenesis pathway, and relied on the host for nutrition of sugar as its main energy income. Other study also suggested on the sugar transportation between Cryptosporidium and human intestine epithele cells[22]. Such transportation would scavenge the substrate gaining of host cells in glycolysis, and may correspondingly lead to the down-regulation of the host genes in the glycolysis subpathway. The Pentose phosphate pathway and Fructose and mannose metabolism were upper or downstream pathways of Glycolysis / Gluconeogenesis, which may participate in the parasite most important energy producing process.

Metabolic pathways which were categorized as lipid metabolism were also included in DEGs significantly enriched host-parasite common subpathways. According to the study of Zhu and Fritzler, de novo biosynthesis of fatty acids probably did not occur in C. parvum [23,24]. But C. parvum possessed three fatty acyl synthases (ACS). These enzymes activated fatty acids by converting them into fatty acyl-CoAs, and in some systems these same proteins can function in lipid trafficking and import [24].

Purine biosynthesis and amino acid metabolic pathways, including Alanine, aspartate and glutamate metabolism, and Glycine, serine and threonine metabolism did not exist in C. parvum either. The metabolites were imported from hosts instead, which is a common feature of apicomplexans. It is worth noting that these pathways were all detected as significant according to our results.

To further examine the regulation pattern of ortholog and non-ortholog genes in host cells, we examined the expression state of each subpathway as we did in Plasmodium, but throughout different time points. Results are shown in Table 2, which provides the number of pathways that were found significant in any of the states. As the data indicates, besides infection after 6 hours, a small number of pathways had a significant number of ortholog genes up-regulated.

**Table 2:** Significantly altered sub-pathways at different time points after infection of Cryptosporidium

Time post infection	Common.up	Common.down	Specific.up	Specific.down
6 hours	18	9	17	9
12 hours	1	7	12	12
24 hours	2	7	9	6
48 hours	0	13	6	13
72 hours	7	23	9	14

Number of significantly altered sub-pathways at each time point (adjusted p –value≤0.05)

## Discussion

From the analysis of gene expression of Plasmodium infected cells, we found the host-parasite common subpathway are more likely to present a down-regulation pattern, as there is only one subpathway that is enriched with up-regulated genes. Moreover, subsequent analysis indicates this phenomenon also existed in Cryptosporidium parvum infected human cells. We performed Wilcoxon signed rank test between the number of up- and down-regulation genes enriched host parasite common subpathways, the result was significant (p-value= 0.02726). Here we did not include the dataset of infection after 6 hours which is still in the invasion stage, and different from others.

Something interesting in the cells infected by the Plasmodium is that the number of pathways having a down-regulated pattern on ortholog genes grew with time, while the same manner appeared in the pathways with a significant alteration in ortholog genes. We excluded the result of 6 hours after infection which was still in the invasion stage, and Spearman correlation test showed positive correlation (correlation coefficient= 0.9486833, p-value= 0.02566, one-sided test), indicating that as infection progressed, more host-parasite common subpathways were impacted, which displayed down-regulation pattern on enzymes or compounds in the corresponding subpathways. However, more samples at different times after infection are needed to further test and verify this trend.

One factor that caused the down-regulation of host-parasite common subpathways in the metabolic pathways was competition of metabolites by the parasite. The gene in fatty acid metabolism pathway (KEGG ID hsa00071) which encodes the acyl-CoA synthetase long-chain family member 1 (EC: 6.2.1.3) was significantly down-regulated. Previous study suggested that the enzymes encoded by ortholog of plasmodium, long chain fatty acid ligase (EC: 6.2.1.3), would enter the host cytoplasm through the apicomplex and was involved in the formation of Hexadecanoyl-CoA [4,5]. The activity of parasite enzyme also activated the downstream formation of CoA (see Figure 2), which would cause downstream genes in the host-specific subpathway significantly up-regulated.

The host-specific subpathways might tend to have a up-regulate pattern because of the interference of parasite enzyme in the host-parasite common subpathways, as was shown in the numbers of host-specific subpathways in Plasmodium infected cells: 12 subpathways were enriched with up-regulated genes and 4 were enriched with down-regulated genes. This assumption could not be fully proved from the analysis of C. parvum (p-value = 0.5724). The expression pattern of host specific subpathways was rather complex as genes in these pathways were under complicated regulations. The topology of these subpathways may also contribute to the complexity: some of them are up-stream of the host-parasite common subpathways and some have parallel host-specific subpathways. Another reason for the variation to predict nutrition relationship between host and parasite might be due to the incompleteness of the pathways from which we retrieve the topological information. Because the KEGG pathway was structured mainly on the genome annotation from the sequence data of a few species, unavailablely there might be inaccuracy associated with some particular pathway. There would be gene merely similar at the sequence level, but it may not necessarily have the same function as the orthologs in other species. According to the gene annotation of KEGG database, a large number of putative genes were found in the KEGG metabolic pathway of these two species: Plasmodium falciparum and Cryptosporidium parvum. Care must be taken in metabolic pathways analysis, and more study needs to be done with updated pathway information.

## Conclusions

In this study, we introduced a new method for exposing the interactions between infected host cells and parasites: Plasmodium falciparum and Cryptosporidium parvum. We divided the host metabolic pathway into two parts: host specific subpathways and host-parasite common subpathways according to ortholog gene information of host and parasite. We performed analysis on the subpathways separately, and the results showed different character between these two groups subpathways. With this method, we revealed the impact of parasite infection on host cell gene expression, which was previously concealed in the pathway enrichment analysis. We provided the results from statistical analyses and visualized them by mapping the data to the KEGG pathway. Our approach revealed detailed subpathways and metabolic information that are important to the symbiosis in two kinds of the apicomplex parasites. The results highlight the significance of our approach in research and understanding of the interactions between parasites and host cells.

## Competing interests

The authors declare that they have no competing interests.

## Authors' contributions

TX ,PJ and PH conceived the study. TX and YY performed statistical analyses for the study, and drafted the manuscript. FY and YT advised on the analytical methods. XL directed the study, and reviewed and finalized the revised manuscript. All authors approved the final manuscript.

## Supplementary Material

Additional file 1 Analysis results of gene expression data of Plasmodium infected human cell.
The file “Additional file_Plasmodium.xls” contains expression level of genes and used 0/1 to indicate if they were DEGs. The table also shows the significantly up- or down- regulated host-parasite common or host specific subpathways according to the enrichment result. Click here for file

Additional file 2 Analysis results of gene expression data of C.parvum infected human cell.
The file “Additional file_Cparvum.xls” contains differentially expressed gene list and the corresponding expression level of the genes. It also shows significantly up- or down-regulated host-parasite common or host specific subpathways in different time points. Click here for file

Additional file 3 Significantly altered pathways in C.Parvum infected human cell.
It contains the significantly altered pathways after infection of C.Parvum. Click here for file
